# Investigating the Prehistory of Tungusic Peoples of Siberia and the Amur-Ussuri Region with Complete mtDNA Genome Sequences and Y-chromosomal Markers

**DOI:** 10.1371/journal.pone.0083570

**Published:** 2013-12-12

**Authors:** Ana T. Duggan, Mark Whitten, Victor Wiebe, Michael Crawford, Anne Butthof, Victor Spitsyn, Sergey Makarov, Innokentiy Novgorodov, Vladimir Osakovsky, Brigitte Pakendorf

**Affiliations:** 1 Department of Evolutionary Genetics, Max Planck Institute for Evolutionary Anthropology, Leipzig, Germany; 2 MPRG on Comparative Population Linguistics, Max Planck Institute for Evolutionary Anthropology, Leipzig, Germany; 3 Department of Anthropology, University of Kansas, Lawrence, Kansas, United States of America; 4 Research Centre for Medical Genetics, Russian Academy of Medical Sciences, Moscow, Russian Federation; 5 Institute of Foreign Philology and Regional Studies, North-Eastern Federal University, Yakutsk, Russia; 6 Institute of Health, North-Eastern Federal University, Yakutsk, Russia; University of Cambridge, United Kingdom

## Abstract

Evenks and Evens, Tungusic-speaking reindeer herders and hunter-gatherers, are spread over a wide area of northern Asia, whereas their linguistic relatives the Udegey, sedentary fishermen and hunter-gatherers, are settled to the south of the lower Amur River. The prehistory and relationships of these Tungusic peoples are as yet poorly investigated, especially with respect to their interactions with neighbouring populations. In this study, we analyse over 500 complete mtDNA genome sequences from nine different Evenk and even subgroups as well as their geographic neighbours from Siberia and their linguistic relatives the Udegey from the Amur-Ussuri region in order to investigate the prehistory of the Tungusic populations. These data are supplemented with analyses of Y-chromosomal haplogroups and STR haplotypes in the Evenks, Evens, and neighbouring Siberian populations. We demonstrate that whereas the North Tungusic Evenks and Evens show evidence of shared ancestry both in the maternal and in the paternal line, this signal has been attenuated by genetic drift and differential gene flow with neighbouring populations, with isolation by distance further shaping the maternal genepool of the Evens. The Udegey, in contrast, appear quite divergent from their linguistic relatives in the maternal line, with a mtDNA haplogroup composition characteristic of populations of the Amur-Ussuri region. Nevertheless, they show affinities with the Evenks, indicating that they might be the result of admixture between local Amur-Ussuri populations and Tungusic populations from the north.

## Introduction

Evenks and Evens are spread over a wide area of northern Asia from the Yenissey river in the west to the Chukotka and Kamchatka peninsulas in the east, and from the Taimyr Peninsula in the north to northern China in the south. They are linguistically and culturally closely related with a traditional life-style of highly nomadic hunting and gathering and reindeer herding; their languages belong to the North Tungusic branch of the Tungusic language family [1]. Other Tungusic-speaking groups are settled to the southeast of the Evenks and Evens, along the lower Amur and Ussuri rivers, as well as on Sakhalin island. These include the linguistically closely related Negidal, whose North Tungusic language shows similarities to both Evenki and Even, as well as populations speaking languages classified as South Tungusic, such as the Udegey (also known as Udihe or Udeghe) and Ulchi. In contrast to the Evens and Evenks, the Tungusic peoples of the Amur-Ussuri region, who we here also refer to alternatively as Amur Tungusic, are traditionally sedentary fishermen and hunters rather than nomadic reindeer herders [2,3]. 

Different hypotheses exist concerning the origins of the North Tungusic Evenks and Evens and their relations with the Amur Tungusic peoples. Vasilevič [4] proposed a relatively ancient separation some 3500 years before present (BP) and a split between the Evenks and Evens approximately 1500 years BP, when the North Tungusic groups migrated northwards from an area south of Lake Baikal. In contrast, Tugolukov [5] and Janhunen [6] propose a more recent separation of the North and Amur Tungusic groups some 800 years BP, with the ultimate split of Evenks and Evens possibly occurring as late as the 17^th^ to 18^th^ centuries CE. At this time Turkic-speaking cattle and horse pastoralists, the Yakuts, expanded over the large territory they occupy nowadays [7,8], in the process displacing the Tungusic reindeer herders. During this displacement, the ancestors of current-day Evenks moved west- and northwestwards, while the ancestors of present-day Evens moved to the east and northeast [9].

The spread of the ancestors of the North Tungusic groups over the large territory they occupy nowadays may have been accompanied by different degrees of intermarriage with local inhabitants. Thus, it is assumed that Yukaghir groups were assimilated by Evenks and especially Evens [9], a process that has continued until recent times [10]. Nowadays, the settlement pattern of these North Tungusic populations is highly fragmented, with small communities living interspersed with other peoples, such as Kets and Samoyedic groups in the west, Buryats in the southwest, Yakuts and Yukaghirs in the central regions, and Chukchi and Koryaks in the east. This has led to a large degree of dialectal diversification of both the Evenki and Even language [11,12], possibly due to contact with the languages spoken by their neighbours. The South Tungusic populations, on the other hand, live in the vicinity of the Nivkh and formerly of the Ainu. The Nivkh speak an isolate language and were traditionally fishermen and hunters of sea mammals [13].

The populations of northern Asia are characterized in the maternal line by high frequencies of mtDNA haplogroups C and D [10,14–18]; in contrast, the peoples of the Amur-Ussuri region carry high frequencies of haplogroups Y1 and N9b [10,18,19], while the peoples of Kamchatka are characterized by high frequencies of haplogroup G1, also common in the Negidal [18,20]. In the paternal line, Y-chromosomal haplogroup C is widespread over a large area encompassing both Siberia and the Amur-Ussuri region, being found in high frequency in North Tungusic and Amur Tungusic populations as well as in the Nivkh. In contrast, northern Siberian populations are characterized by high frequencies of haplogroup N, with N1c being the predominant haplogroup found in the Yakuts [14,15,17,19,21–26]. There are thus discrepancies between the maternal, paternal, and linguistic perspectives concerning the population history of northern Asia and the Amur-Ussuri region: whereas the mtDNA data point to an ancient divergence between peoples inhabiting the two regions, the Y-chromosomal haplogroup frequencies link the Tungusic peoples of central and northeastern Siberia with those of the Amur-Ussuri area, and linguistically the Evenks, Evens, Negidals, and South Tungusic populations such as the Udegey and Ulchi share a relatively recent common ancestor.

The historical expansion and the resulting fragmented settlement pattern of the Evenks and Evens raises the question to what extent different communities have intermarried with their geographic neighbours rather than their linguistic and cultural relatives. A previous investigation into the relationship of Evenks and Evens found evidence for shared ancestry in both the mtDNA and Y-chromosomal data, with subsequent isolation of men belonging to different subgroups as well as indications of intermarriage with neighbouring populations in the maternal line [15]. In this study, we refine our analysis with complete mtDNA genome sequences for four subgroups of Evenks and five subgroups of Evens in comparison to their linguistic relatives, the South Tungusic Udegey, as well as to their geographic neighbours the Yakuts, Yukaghirs, and Koryaks. We also include the geographic neighbours of the Udegey, the Nivkh, to shed light on the question to which extent admixture rather than shared ancestry has shaped the genepool of the populations of Siberia and the Amur-Ussuri region. To explore the paternal relationships of the North Tungusic groups, Y-chromosomal haplogroup and short tandem repeat (STR) data are taken into account. With this data set, we are able to demonstrate that genetic drift and differential admixture have attenuated the signal of shared ancestry among the North Tungusic populations, and that the Udegey are likely to be the result of admixture in the maternal line between indigenous Amur-Ussuri populations and Siberian populations presumably speaking Tungusic languages.

## Results

### MtDNA diversity and haplogroup composition

The populations of the Amur-Ussuri region (Udegey and Nivkh) are characterized by low sequence diversity (0.88) and intermediate and low nucleotide diversity values (0.0016 and 0.0011, respectively; Table 1). This contrasts with the Yakuts, who are among the populations studied here with the highest sequence and nucleotide diversity (0.98 and 0.0022-0.0023, respectively). The Sebjan and Kamchatkan Evens and the Stony Tunguska Evenks have relatively low sequence (0.86, 0.90, and 0.94, respectively) and intermediate nucleotide diversity values (0.0016, 0.0018, and 0.0015, respectively), which contrast with the Sakkyryyr Evens, whose sequence and nucleotide diversity are as high as those found in the Yakuts (0.98 and 0.0023). 

**Table 1:** mtDNA diversity values in the populations studied here with their linguistic affiliation and rough geographic location.

**Population**	**abbrev**	**linguistic affiliation**	**geography**	**N**	**n**	**S**	**Seq div**	**Seq div SD**	**π**	**π SD**
**all Evenk**		**N. Tungusic**	**Siberia**	**130**	**53**	**287**	**0,98**	**0,00**	**0,0020**	**0,0010**
Taimyr	TAI	N. Tungusic	Siberia	24	16	145	0,95	0,03	0,0020	0,0010
Stony Tunguska	STE	N. Tungusic	Siberia	39	17	124	0,94	0,02	0,0015	0,0007
Nyukzha	NYUK	N. Tungusic	Siberia	46	25	175	0,97	0,01	0,0021	0,0011
Iengra	IENG	N. Tungusic	Siberia	21	13	107	0,96	0,02	0,0017	0,0009
**all Even**		**N. Tungusic**	**Siberia**	**122**	**54**	**232**	**0,98**	**0,00**	**0,0020**	**0,0010**
Sakkyryyr	SAK	N. Tungusic	Siberia	23	19	150	0,98	0,02	0,0023	0,0012
Sebjan	SEB	N. Tungusic	Siberia	18	8	71	0,86	0,06	0,0016	0,0008
Tompo	TOM	N. Tungusic	Siberia	27	16	128	0,95	0,02	0,0018	0,0009
Berezovka	BER	N. Tungusic	Siberia	15	11	85	0,95	0,04	0,0017	0,0008
Kamchatka	KAM	N. Tungusic	Kamchatka	39	13	103	0,90	0,02	0,0018	0,0009
**Udegey**	**UDI**	**S. Tungusic**	**Amur-Ussuri**	**31**	**14**	**96**	**0,88**	**0,05**	**0,0016**	**0,0008**
**all Yakut**		**Turkic**	**Siberia**	**169**	**94**	**446**	**0,98**	**0,00**	**0,0023**	**0,0011**
Vilyuy	VIL_YAK	Turkic	Siberia	49	35	252	0,98	0,01	0,0022	0,0011
Central	CNT_YAK	Turkic	Siberia	88	64	368	0,98	0,01	0,0023	0,0011
Northeast	NE_YAK	Turkic	Siberia	32	24	185	0,98	0,01	0,0023	0,0011
**Yukaghir**	**YUK**	**isolate**	**Siberia**	**20**	**13**	**105**	**0,94**	**0,03**	**0,0018**	**0,0009**
**Koryak**	**KOR**	**Chukotko-Kamchatkan**	**Kamchatka**	**15**	**8**	**75**	**0,90**	**0,05**	**0,0018**	**0,0009**
**Nivkh**	**NIV**	**isolate**	**Amur-Ussuri**	**38**	**14**	**60**	**0,88**	**0,04**	**0,0011**	**0,0006**

abbrev = abbreviation used in figures and tables; N = sample size; n = number of haplotypes; S = number of polymorphic sites; Seq div = sequence diversity; SD = standard deviation; π = nucleotide diversity

**Figure 1:**
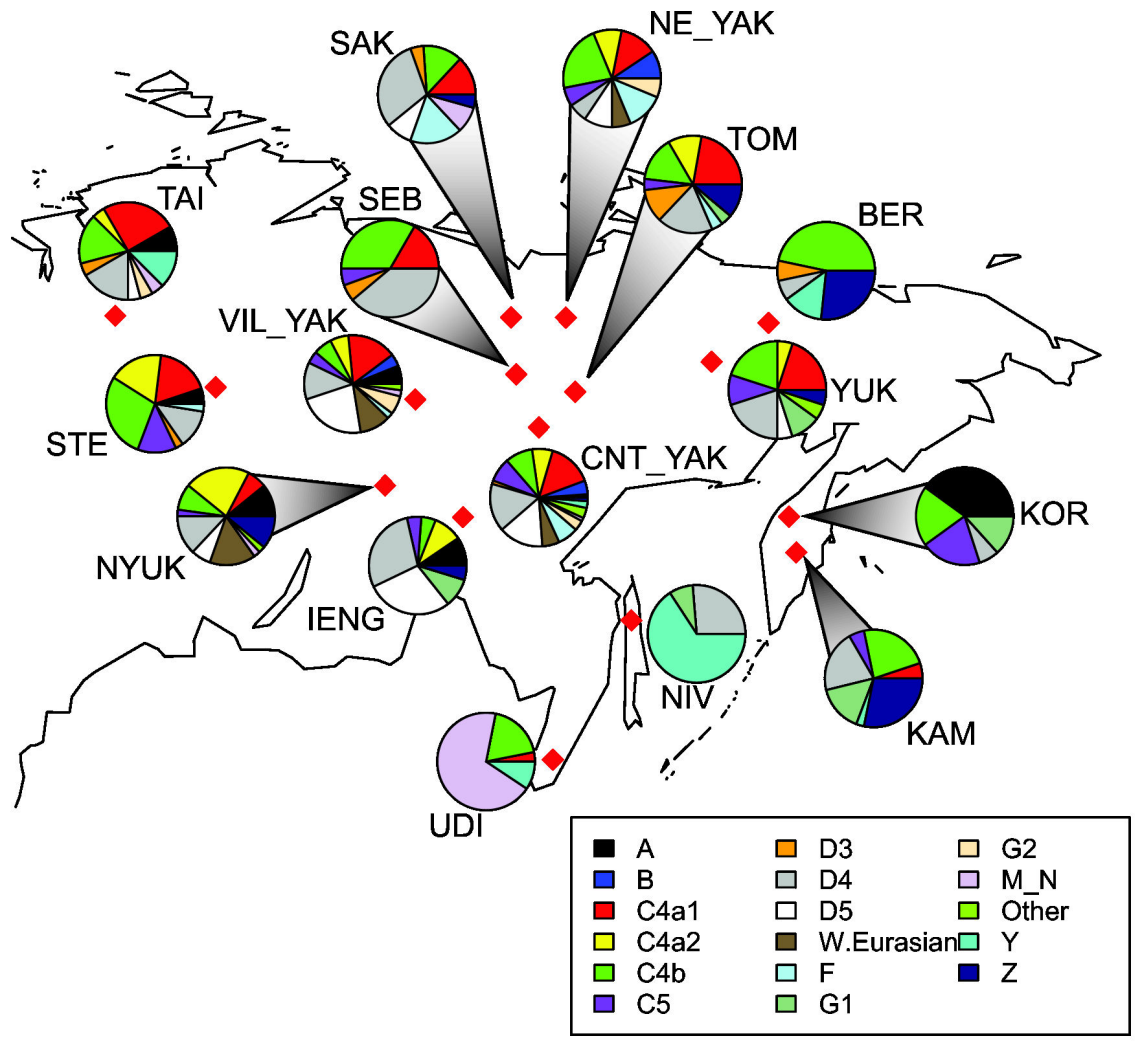
Map of Siberia showing approximate locations of sampled populations and their basic haplogroup composition. (Sub)population abbreviations as in Table 1.

As can be seen in Figure 1 and Table S1, haplogroups D4, C4b, and C4a1 are the most frequent haplogroups among the populations analysed here (16.3%-11.4% overall; cf. [17]). Although the Evens and Evenks are characterized by high frequencies of these common haplogroups, they also differ in their haplogroup composition: Evens have a much higher frequency of haplogroup Z (15.6%) than Evenks (4.6%), while Evenks have a much higher frequency of C4a2 (15.4%) than Evens (2.5%), as well as of haplogroup A (8.5%), which is absent among the latter. Subhaplogroup C4b3a is present only in Evens and Yukaghirs, as found previously [17]; in contrast, subhaplogroup C5d1, which was previously suggested to be common in Evens and Yukaghirs [17], is found in four Stony Tunguska Evenks as opposed to only one Yukaghir and two Evens (Table S1). Furthermore, the subhaplogroup of C4b carrying the T3306C transition defined by Fedorova et al. [17] as C4b9, which they suggest is common in Evenks, is found here only in Evens (belonging to the Sakkyryyr and Sebjan subpopulations).

Differences exist among the individual Evenk and Even subgroups, too (Table S1). For instance, two sequences belonging to haplogroup A2a are found among the Taimyr Evenks, but not in any of the other Tungusic or neighbouring populations. Haplogroup D5a2a2, which is frequent in Yakuts, is found in nearly 29% in the Iengra Evenks and nearly 9% in the Sakkyryyr Evens, but is absent in the Stony Tunguska Evenks and other Even subgroups. Among the Evens, haplogroup Z is found at particularly high frequency in the eastern subgroups Kamchatka (28.2%) and Berezovka (26.7%) and in intermediate frequency in the Tompo Evens (11.1%), but is low to absent in Sakkyryyr and Sebjan, respectively. All the individuals classified here as Z1a carry the A11252G transition and thus correspond to what was called Z1a3 by Fedorova et al. [17], while all individuals classified in our study as Z1a1 carry the G7521A and G8251A mutations and thus correspond to what they defined as clade Z1a1b. 

The South Tungusic Udegey, who are the linguistic relatives of the Evenks and Evens, have a very different haplogroup composition (Table S1): they lack D4, exhibit only low frequencies of C4a1, and have high frequencies of haplogroups N9b (32.3%), M7a2a (16.1%), and M9a1 (9.7%). Other than one M7a2a sequence in the Nyukzha Evenks, these haplogroups are not found in any of the other populations studied here. In addition, the Udegey have 12.9% of haplogroup M8a1 which was not previously recorded in populations of Siberia or the Amur-Ussuri region. In their haplogroup composition, the Udegey also differ from their neighbours the Nivkh, who have 65.8% haplogroup Y1a and 26.3% D4m2, a haplogroup found in low frequencies in Evens and Yakuts, but lacking in the Udegey (Table S1).

### MtDNA haplotype sharing analyses and networks

An analysis of shared haplotypes (Table S2) demonstrates that most of the sharing involves the different Yakut subgroups: 35 of the 54 haplotypes that are shared across different (sub)populations involve Yakuts, and 14 are shared only among different Yakut subgroups. Five haplotypes are shared only between Evenks and Yakuts, and five others are shared only between Evens and Yakuts. All five of the haplotypes shared solely between Evens and Yakuts are found in the Sakkyryyr Evens, and four are shared exclusively between Sakkyryyr Evens and Yakuts, demonstrating the considerable amount of admixture in the maternal line that has taken place between this Even subgroup and the Yakuts. Four haplotypes are shared only between Evens and Yukaghirs, while none are shared solely between Evenks and Yukaghirs, which is in good accordance with the greater geographic proximity of the Yukaghirs and the Evens. Three haplotypes are shared exclusively between Evenks and Evens; this contrasts with six haplotypes shared only among Evenk subgroups and four shared only among Evens. The Udegey, Nivkh, and Koryaks each share only one sequence with another population included here, although there is considerable haplotype sharing within these populations (Table S2). The analysis of haplotype sharing thus demonstrates the distinctiveness of the populations of the periphery (Amur-Ussuri region and Kamchatka), as also reflected in their divergent haplogroup composition (Table S1), a certain level of divergence between the Evenks and Evens, and the central position played by the Yakuts, who share haplotypes with Evenks, Evens, Yukaghirs, and even Nivkh and Udegey.

**Figure 2:**
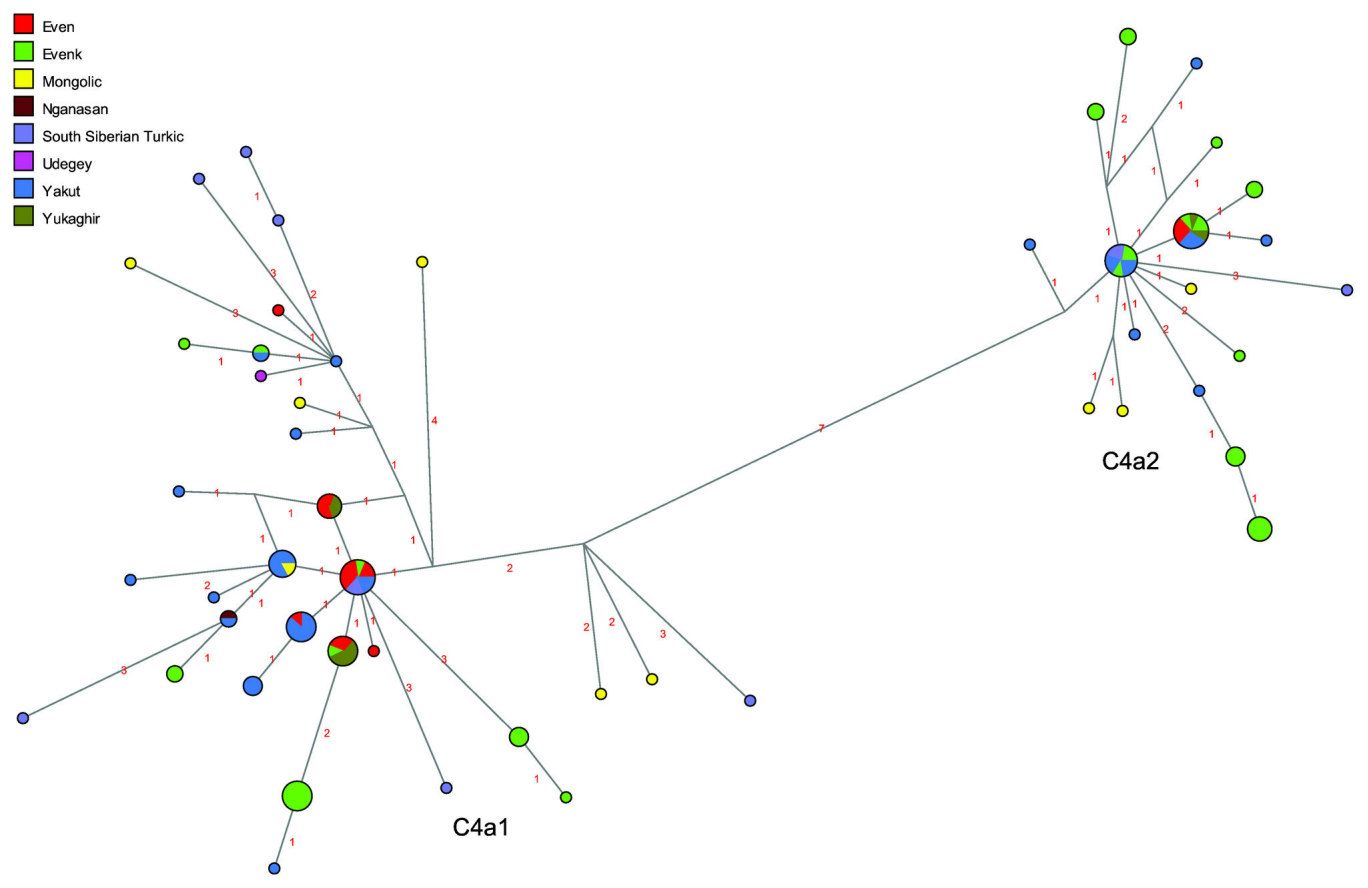
MJ-network of mtDNA haplogroup C4a. The North Tungusic haplotypes are coloured by population (Evenks and Evens) rather than subgroup. The size of the nodes is proportional to the number of individuals carrying that node, and the number of mutations is indicated along the branches. The labelled subhaplogroups are discussed in the text.

**Figure 3:**
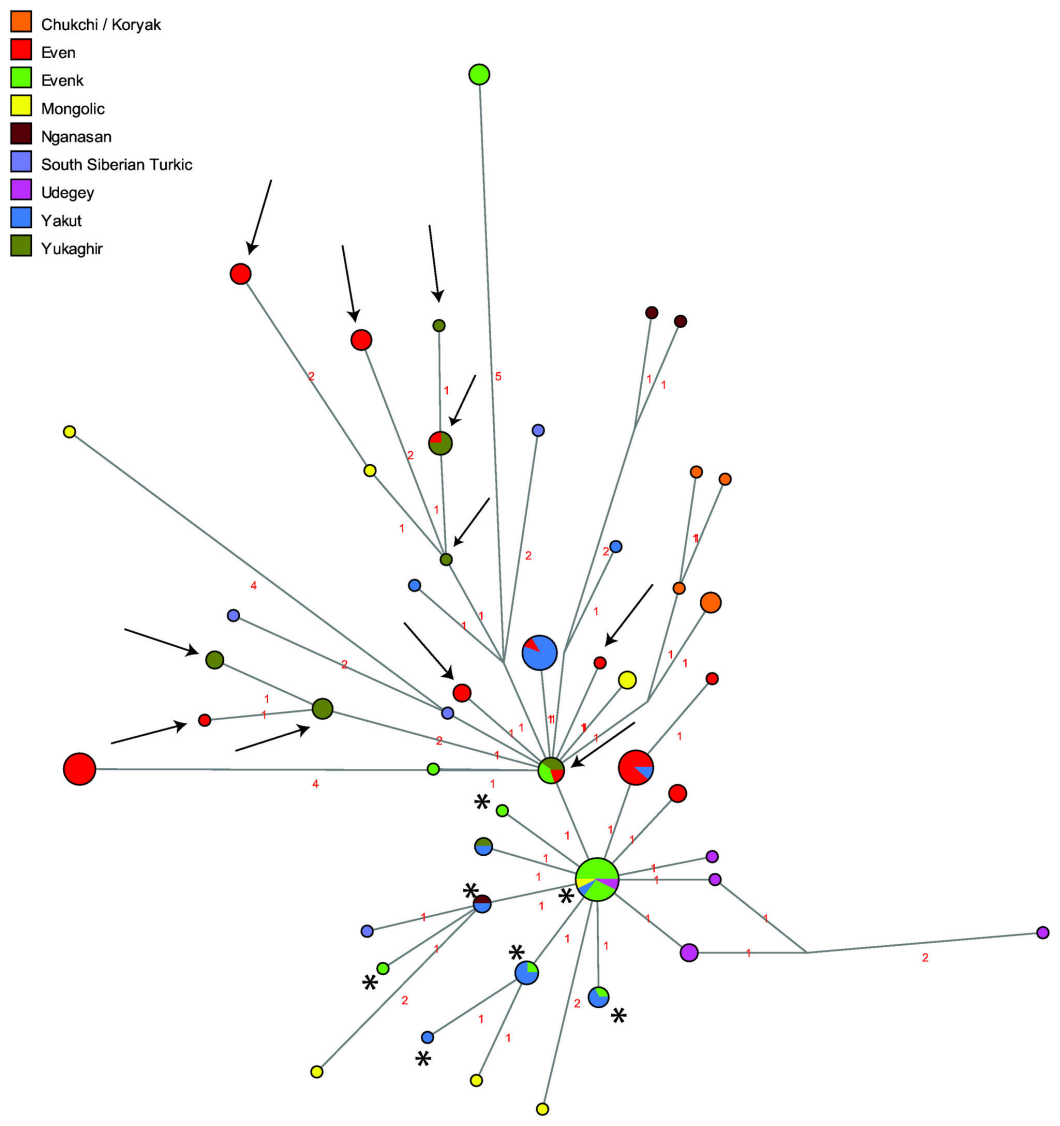
MJ-network of mtDNA haplogroup C4b. The North Tungusic haplotypes are coloured by population (Evenks and Evens) rather than subgroup. The size of the nodes is proportional to the number of individuals carrying that node, and the number of mutations is indicated along the branches. The haplotypes marked by arrows and asterisks are discussed in the text.

Analyses of haplotype sharing only provide information on identical sequences shared between populations; network analyses in addition show closely related, albeit not identical, sequences. Note, however, that the results of the two types of analysis are not entirely comparable, as they are based on different alignments, as described in the Material & Methods section below. The network of C4a (Figure 2) shows that whereas subhaplogroup C4a1 is widespread in Siberian populations, subhaplogroup C4a2 is dominated by Evenk and Yakut sequences. Thus, of 18 haplotypes, nine are found in Evenks and seven are found in Yakuts; only one haplotype, which is shared with Evenks, Yakuts, and Yukaghirs, is found in Evens. The network of haplogroup C4b (Figure 3) further illustrates the proximity of Evenks and Yakuts, who tend to fall upon the same branches with only one mutation between haplotypes (shown by asterisks in the figure), while the Evens tend to share branches with the Yukaghirs (indicated by arrows in the figure), confirming the genetic proximity of these populations that emerged in the analysis of shared haplotypes. This network also demonstrates that the Udegey C4b sequences originate from a central haplotype shared with several Evenks as well as a Yakut and a Buryat; haplotypes found in Evens also derive from this central node. 

**Figure 4:**
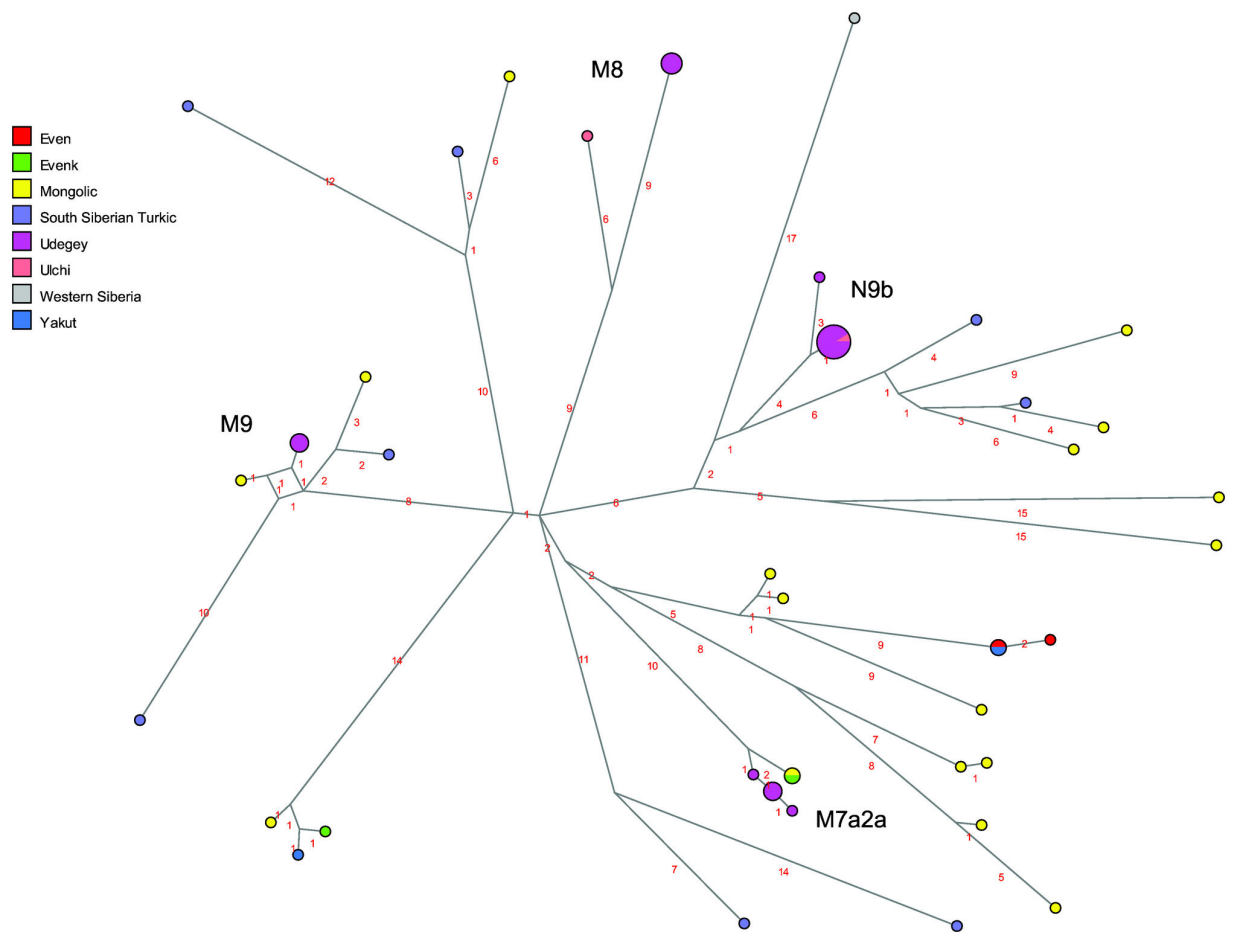
MJ-network of various mtDNA M and N subhaplogroups. The North Tungusic haplotypes are coloured by population (Evenks and Evens) rather than subgroup. The size of the nodes is proportional to the number of individuals carrying that node, and the number of mutations is indicated along the branches. Subhaplogroups discussed in the text are labelled.

The network of different M and N subhaplogroups (Figure 4) clearly illustrates the distinctiveness of the Udegey, and suggests different affinities of the sequence types found in this population. The sequences belonging to subhaplogroups M9 and M7a2a are relatively close to sequences found in southern Siberian populations (Nyukzha Evenks, Buryats, Khamnigan, and Tuvan), while the M8 and N9b sequences are quite distinct, with only one sequence found in the Ulchi, another South Tungusic population of the Amur-Ussuri region, being shared with the largest Udegey N9b haplotype. 

The network analyses also provide evidence of geneflow. The network of haplogroup D4 (Figure S1) demonstrates that the D4o2 sequence shared between Koryaks and Kamchatkan Evens (indicated by arrow 1 in the figure) is more likely to be due to gene flow from Kamchatkan Evens into Koryaks than the other way round, since haplogroup D4 is not at all characteristic of the populations of the Chukotka and Kamchatka Peninsulas. This network furthermore demonstrates that the Nivkh sequences belonging to subhaplogroup D4m2 stem from a haplotype shared with Yakuts (indicated by arrow 2 in Figure S1) and belong to the same branch as sequences found in Evenks, Evens, Yukaghirs, and South Siberian Buryats and Turkic speakers – all populations settled far to the north or west of the Nivkh. The network of D5a2 (Figure S2) demonstrates the effect of Yakut gene flow into neighbouring Tungusic populations: sequences belonging to this haplogroup are found especially in the Iengra Evenks, but are lacking from the Stony Tunguska Evenks, who are the most distant geographically from the Yakuts. Among the Even subgroups, it is found only in the Sakkyryyr Evens, a group that has already largely lost its indigenous Even language and now speaks Yakut.

The haplogroup A sequences found in the Stony Tunguska, Nyukzha, and Iengra Evenks, which belong to haplogroup A4, fall with other haplogroup A4 sequences from southern and western Siberian populations (Figure S3). They thus form part of the general geographic region of their provenance, although several mutations separate the Evenk haplotypes from those found in other populations. In contrast, the two Taimyr Evenk individuals, who carry an identical haplogroup A2a sequence, cluster with Koryak, Chukchi, and Eskimo individuals. Even though there is no direct sharing, only one mutation separates this haplotype from a sequence type found in Eskimo. In a previous study based only on HVR1 sequences, a Yakut-speaking Evenk from northwestern Yakutia (in the vicinity of the Taimyr Peninsula) was also found to carry an A2 sequence [14]. This is notable given the great geographic distances separating these populations. 

### Maternal population structure and genetic differentiation

As shown by the AMOVA analysis (Table 2), 9.5% of the total variance in the dataset is due to between-population differences. The proportion of the variance due to differences between four basic geographic groups (west, central, northeast, southeast) is smaller than the variance among the populations grouped by this criterion (4% vs. 6.3%), indicating that the geographic location of the populations has not had a major effect in shaping their genetic variation. This might be due to the extreme mobility of the nomadic North Tungusic groups. In contrast, grouping the populations by linguistic affiliation appears to provide a better fit to the data, with 6.7% of the variance being found between linguistic groups as compared to 4.5% between populations within groups. However, it should be noted that in this grouping there are four groups composed of only one population each (South Tungusic, Chukotko-Kamchatkan, Yukaghir, and Nivkh) – which excludes between-population variance – and is thus not comparable to the grouping based on geography. 

**Table 2:** AMOVA analyses based on mtDNA Φ_ST_ values.

		**percentage of variance**	
**grouping criterion**	**between groups**	**between populations**	**within populations**
**1 group (16 (sub)populations)**		9.47**	**90,53**
4 geographic groups	4.01*	6.29**	89.71**
6 linguistic groups	6,66	4.54**	88.80**
**1 group (Tungusic family)**		8.39**	**91,61**
2 groups (N. Tungusic vs. S. Tungusic)	5,67	6.77**	87.55**
**1 group (North Tungusic)**		7.04**	**92,96**
2 groups (Evenks vs. Evens)	-0,21	7.16**	93.05**
1 group (all Evenks)		8.28**	91,72
1 group (all Evens)		6.00**	94

**4 geographic groups**: West (TAI, STE, NYUK, IENG, VIL_YAK), Central (SAK, SEB, TOM, CNTRL_YAK, NE_YAK), Northeast (BER, KAM, YUK, KOR), Southeast (UDI, NIV)

**6 linguistic groups**: North Tungusic (TAI, STE, NYUK, IENG, SAK, SEB, TOM, BER, KAM), South Tungusic (UDI), Turkic (VIL_YAK, CNTRL_YAK, NE_YAK), Chukotko-Kamchatkan (KOR), Yukaghir, Nivkh

* P-value < 0.05

** P-value < 0.01

When investigating only the Tungusic populations (Evens, Evenks, and Udegey), 8.4% of the variance is due to differences between (sub)populations. Only 5.7% of the variance can be explained by differences between groups when comparing the North Tungusic populations (Evens and Evenks) with the South Tungusic Udegey, with 6.8% of the variance being found between populations in each group. With respect to the North Tungusic populations, 7% of the variation is due to differences between subgroups. That these are due to differences among the individual Evenk and Even subgroups, and not to differences between the Evenk and Even populations, is demonstrated by the non-significant and negative proportion of variance due to between-group differences when comparing Evenks with Evens, while the proportion of between-population variance is highly significant and quite high when comparing only the Evenk subgroups (8.3%) and only the Even subgroups (6%) (Table 2). 

**Figure 5:**
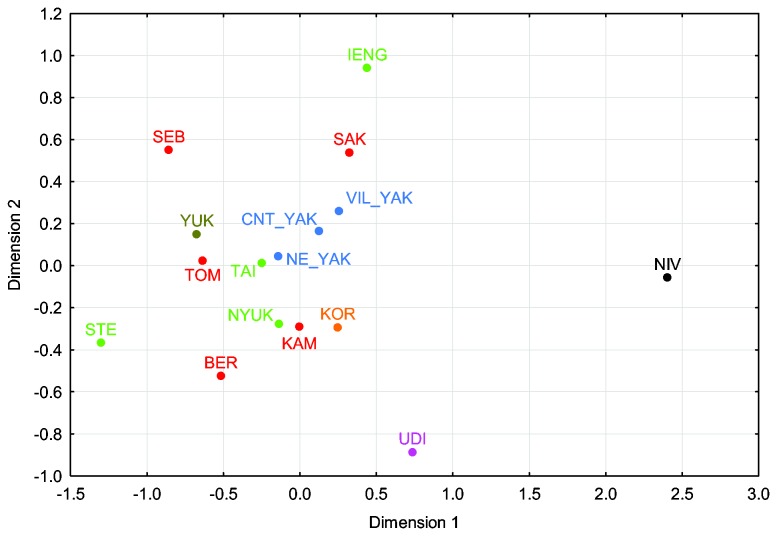
Dimensions 1 vs. 2 of a three-dimensional MDS analysis. Based on pairwise Φ_ST_ values between populations; stress = 0.06. (Sub)population abbreviations as in Table 1, with colours distinguishing the different populations: green = Evenks, red = Evens, pink = Udegey, blue = Yakuts, olive = Yukaghirs, orange = Koryaks, black = Nivkh.

In a three-dimensional MDS plot based on pairwise Φ_ST_ values, the Nivkh and Udegey are the biggest outliers (Figure 5, Figure S4 and Figure S5), with all the other populations clustering fairly closely in the first and second dimension (Figure 5). It is noteworthy that neither the Evenk nor the Even subgroups cluster together in the first or second dimension; in the third dimension, however, the Evenks are in relative proximity to each other. There is geographic clustering among the Even subgroups in the second dimension: the westernmost Sebjan and Sakkyryyr Evens are proximal to each other, as are the easternmost Kamchatkan and Berezovka Evens, and the Tompo Evens are located in an intermediate position. Nevertheless, the Tompo Evens are closer to the Yukaghirs than to other Even subgroups, as has been observed previously [15] (where the Tompo Evens were called Central Evens); the Kamchatkan Evens are close to the Koryaks in the first and second dimension, although the third dimension separates the Koryaks. 

**Figure 6:**
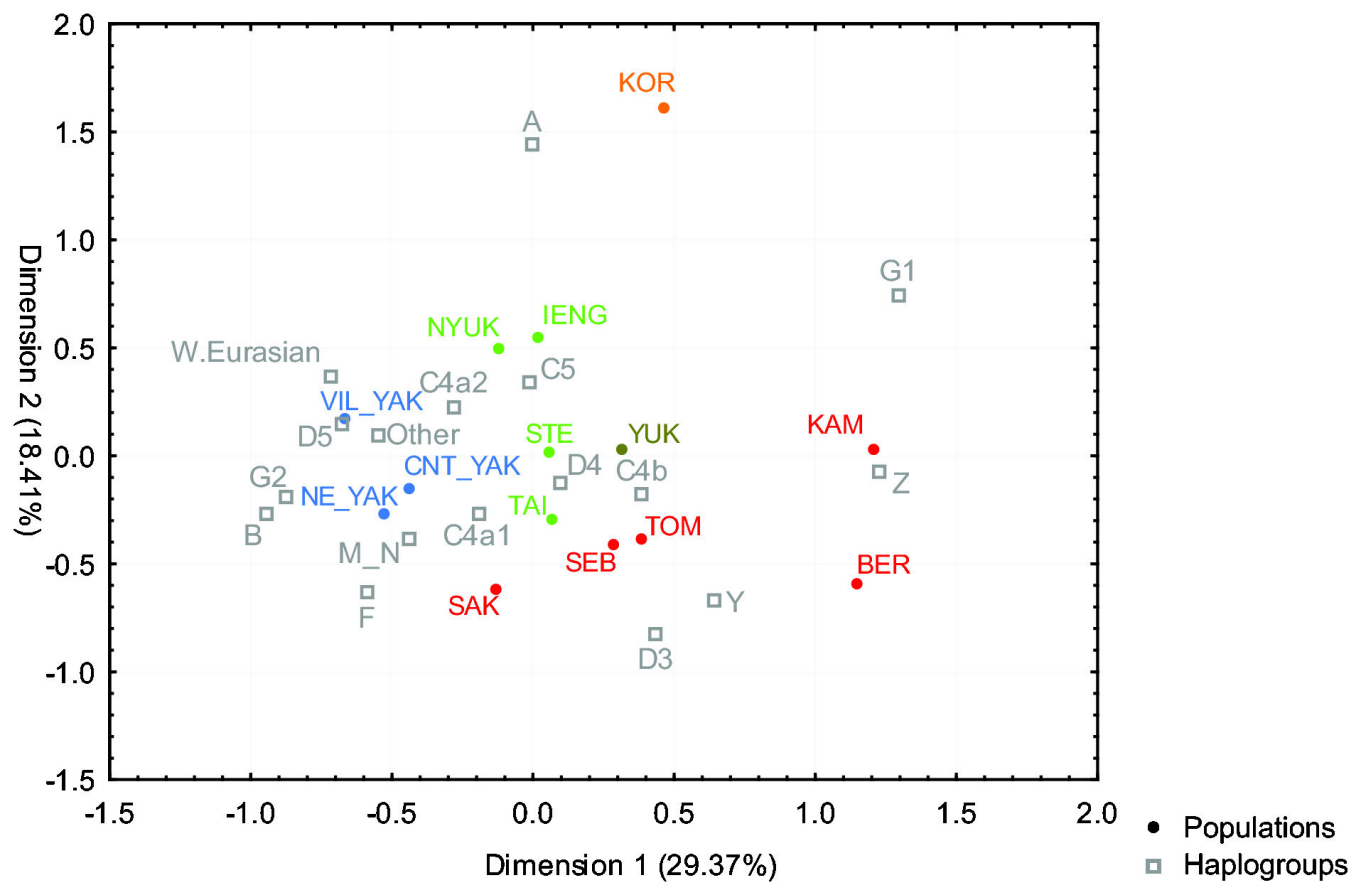
CA plot. Based on mtDNA haplogroup frequencies at the level of resolution depicted in Figure 1, excluding Nivkh and Udegey. (Sub)population abbreviations as in Table 1, with colours distinguishing the different populations: green = Evenks, red = Evens, blue = Yakuts, olive = Yukaghirs, orange = Koryaks; haplogroups in grey.

In a correspondence analysis (CA) based on the frequencies of basic haplogroups at the level of resolution shown in Figure 1, the distinct position of the Nivkh and Udegey due to their high frequencies of haplogroups Y1a and various M and N subgroups, respectively, causes all the other populations to cluster together (not shown). When excluding the Nivkh and Udegey, a central cluster is discernible (Figure 6), with the Koryaks, Berezovka Evens, and Kamchatkan Evens in outlying positions. Although the CA slightly distinguishes clusters of different North Tungusic subgroups, such as the Nyukzha and Iengra Evenks, or the Tompo and Sebjan Evens with the Taimyr Evenks, overall this plot illustrates the common haplogroup composition present in the central Siberian Evens and Evenks. 

A Mantel test of correlations between mtDNA Φ_ST_ and geographic distances reveals that this correlation is not significant when considering all populations (Table 3), in good accordance with the results of the AMOVA analysis. The correlation between the geographic and genetic distances among the Evenk and Even populations is also not significant; neither is the correlation only for the Evenks or Evens. However, the correlation is significant for the Even subgroups when excluding the Sakkyryyr Evens (Table 3), confirming the geographic cline apparent in the MDS plot (Figure S4).

**Table 3:** Results of Mantel tests between mtDNA Φ_ST_ and geographic distances.

**comparison**	**r**	**P**
all populations	0,24	0,12
Evens_Evenks	0,07	0,38
all Evenks	0,12	0,41
all Evens	0,46	0,11
4 Evens (no SAK)	0,71	0,04

### Bayesian Skyline Analysis of mtDNA sequences

**Figure 7:**
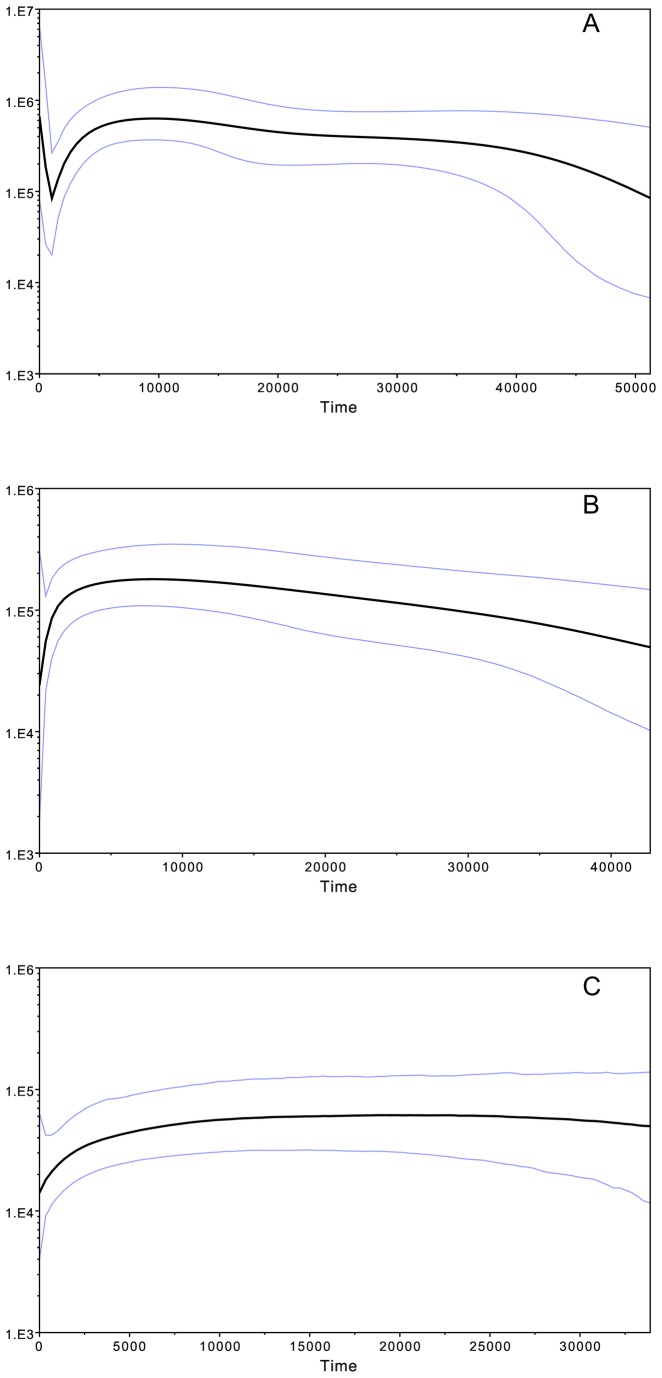
Bayesian Skyline Analysis plots. Based on complete mtDNA genome sequences and a strict clock model. A: All Yakuts; B: All Evens; C: Udegey.

The Bayesian Skyline plots show three different patterns, illustrated here with the Yakuts (Figure 7A), the Evens (Figure 7B), and the Udegey (Figure 7C); the plots for the Evenks, Yukaghirs, Koryaks, and Nivkh are included in the Material (Figure S6). In the Yakuts, there is a notable decline in population size 3000-5000 years ago (ya) followed by a sharp increase ~1000 ya. This contrasts with the Evenks and Evens, where a notable decline in population size 1000-2000 ya is not followed by an increase. The Udegey, Yukaghirs, Nivkh, and Koryaks all have relatively flat curves with at most a gentle decline close to the present. Furthermore, the effective population size of the Yakuts, Evenks, and Evens is larger than that of the Udegey, Yukaghirs, Nivkh, and Koryaks.

### Y-chromosome analyses

As can be seen in Table 4, haplogroup C3c1 (defined by SNP M86) is the most frequent Y-chromosomal haplogroup found in both the Evenks (56.6%) and the Evens (48.3%). As with the mtDNA haplogroup composition, there are notable differences between subgroups. The Taimyr Evenks, Stony Tunguska Evenks, and Tompo Evens carry high frequencies of haplogroup N1b (38.9%, 27.5%, and 42.9%, respectively), which is otherwise found at low frequency only in the Sakkyryyr Evens. Haplogroup N1c, which is highly characteristic of Yakuts [14,17], is found at very high frequency in the two western Even subgroups Sakkyryyr (76%) and Sebjan (64.3%) and in high frequency in the Iengra Evenks (22.2%) and Nyukzha Evenks (20.5%). In contrast, the eastern Even subgroups Berezovka and Kamchatka carry only haplogroup C3c1.

**Table 4:** Y-chromosomal haplogroup frequencies.

**population**	**N**	C*	C3*	C3c*	**C3c1**	F*	I*	J2*	N1b*	**N1c**	O*	Q1*	R1a*	R1a1*	**reference**
**all Evenk**	**127**		**13**	**2**	**82**	**1**	**5**	**1**	**18**	**18**	**1**		**4**		
TAI	18				8				7				3		this study
STE	40				28		1^$^		11						Pakendorf et al. 2006
NYUK	78		13	n/a	42	1	4	1		16			1		Karafet et al. 2002
IENG	9			2	4					2	1^$$^				Pakendorf et al. 2007
**all Even**	**89**	**1**	**1**	**1**	**43**				**13**	**30**					
SAK	25			1	4				1	19					Pakendorf et al. 2007, this study
SEB	14	1			4					9					Pakendorf et al. 2007, this study
TOM	28		1		13				12	2					Pakendorf et al. 2007, this study
BER	7				7										this study
KAM	15				15										this study
**all Yakut**	**184**		3		1			1	1	173		1		4	Pakendorf et al. 2006
**Yukaghir**	**13**		1	1	2	1				4		4			Pakendorf et al. 2006

^$^ note that in the original publication, this STE individual had been erroneously genotyped as belonging to haplogroup F; retyping showed he belonged to haplogroup I-M170

^$$^ note that in the original publication, this IENG indivudal had been erroneously genotyped as belonging to haplogroup K; retyping showed he belonged to haplogroup O-M175

**Figure 8:**
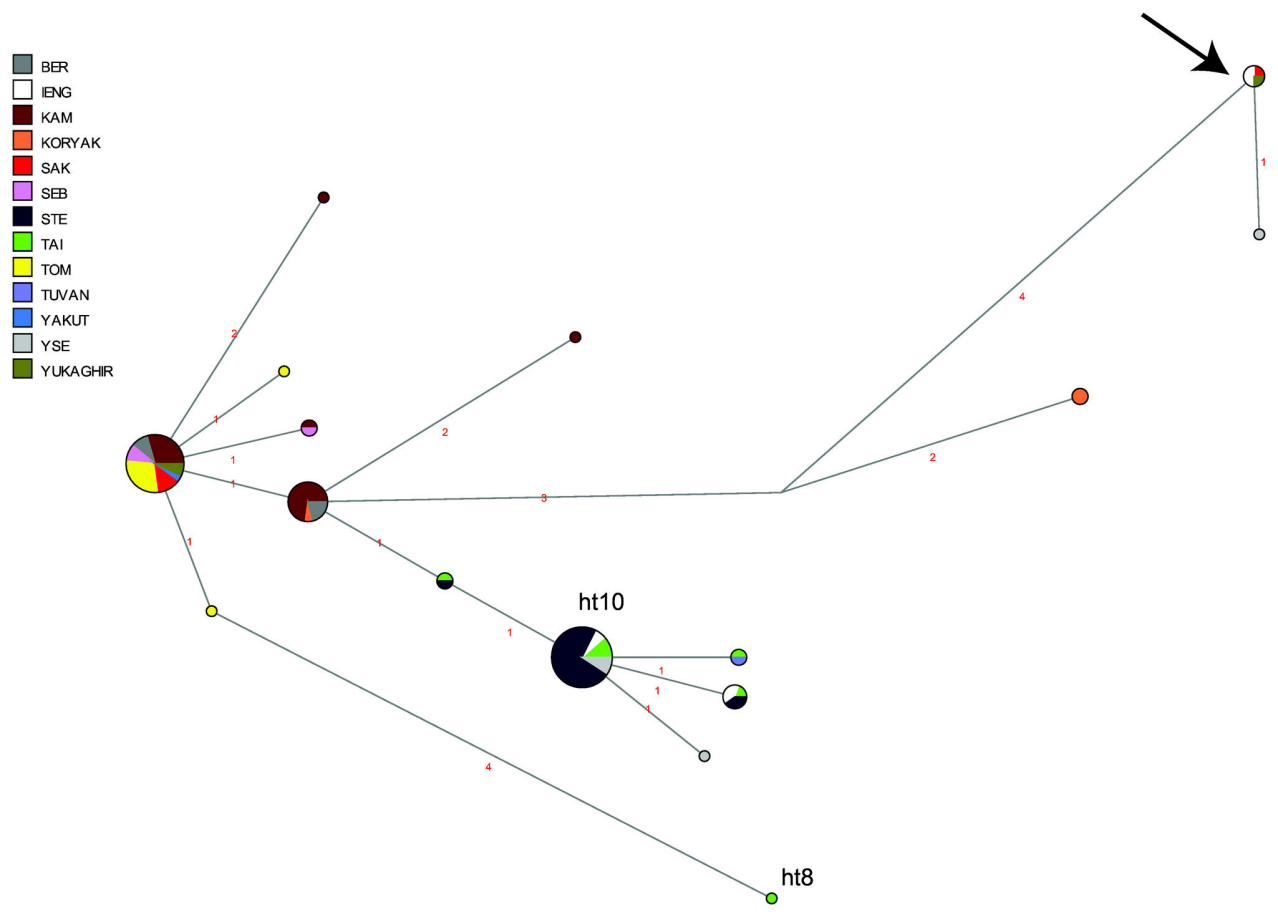
MJ-network of Y-chromosomal haplogroup C3c. Based on nine Y-STRs. The North Tungusic haplotypes are coloured by subgroup. The size of the nodes is proportional to the number of individuals carrying that node, and the number of mutations is indicated along the branches. The haplotype marked by an arrow is discussed in the text. Note that the placement of the Taimyr Evenk haplotype #8 as undertaken by Network is erroneous; as evident from Table S3, this haplotype is only two mutational steps distant from haplotype #10, also labelled in the figure.

In a network of Y-STR haplotypes belonging to haplogroup C3c based on nine STRs and including published data, three major haplotypes are apparent in C3c1 (Figure 8). One of these consists entirely of Evenk individuals from different subgroups as well as Yakut-speaking Evenks from northwest Yakutia, in direct neighbourhood to the Taimyr Peninsula; one consists entirely of eastern Evens (Berezovka and Kamchatka) plus one Koryak individual, and the third consists of Even individuals from all five subgroups plus Yukaghirs and one Northeastern Yakut. Only one haplotype, belonging to subgroup C3c* (defined by the derived state at M48 and the ancestral state at M86, marked by an arrow in Figure 8), is shared between Evenks and Evens (the Iengra and the Sakkyryyr subgroup, respectively) as well as Yukaghirs. This parallels the findings of the mtDNA haplotype sharing analyses, where only three haplotypes were shared between Evenks and Evens that were not also shared with Yakuts or Yukaghirs.

**Figure 9:**
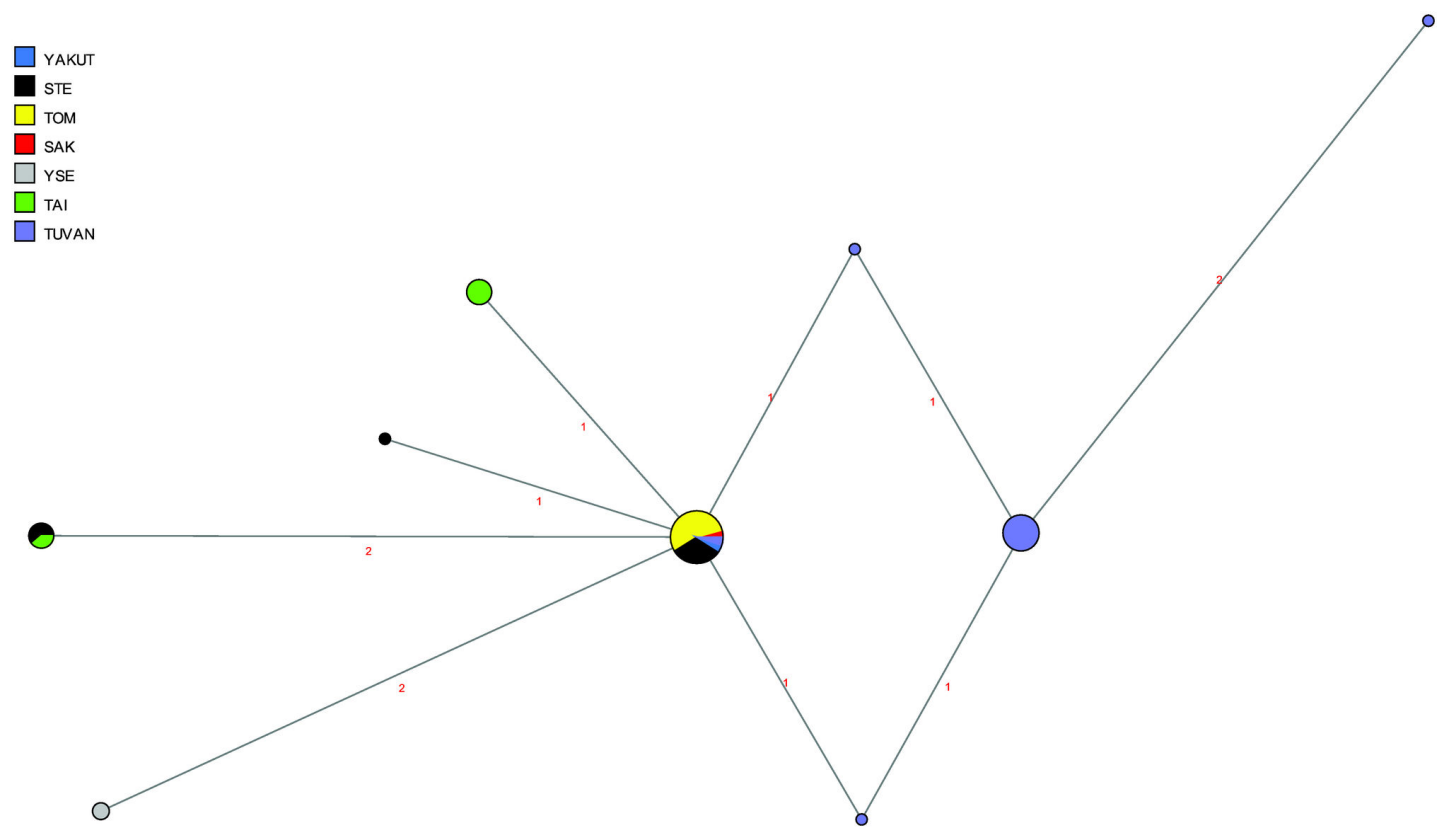
MJ-network of Y-chromosomal haplogroup N1b. Based on nine Y-STRs. The North Tungusic haplotypes are coloured by subgroup. The size of the nodes is proportional to the number of individuals carrying that node, and the number of mutations is indicated along the branches.

As becomes apparent from the network of STR haplotypes belonging to haplogroup N1b (Figure 9), the 13 Evens carry only one haplotype, which is shared with Stony Tunguska Evenks; it is also found in one Yakut and one Tuvan. There is somewhat more diversity in the Evenks, with four different haplotypes, of which three are found in the Stony Tunguska Evenks, and two are found in the Taimyr Evenks; only one haplotype is shared between the two Evenk subgroups. The haplotypes found in the Turkic-speaking Tuvans from southern Siberia are distinct from those found in the Tungusic-speaking Evenks and Evens, with the exception of one individual who carries the haplotype also found in Evenks and Evens.

**Figure 10:**
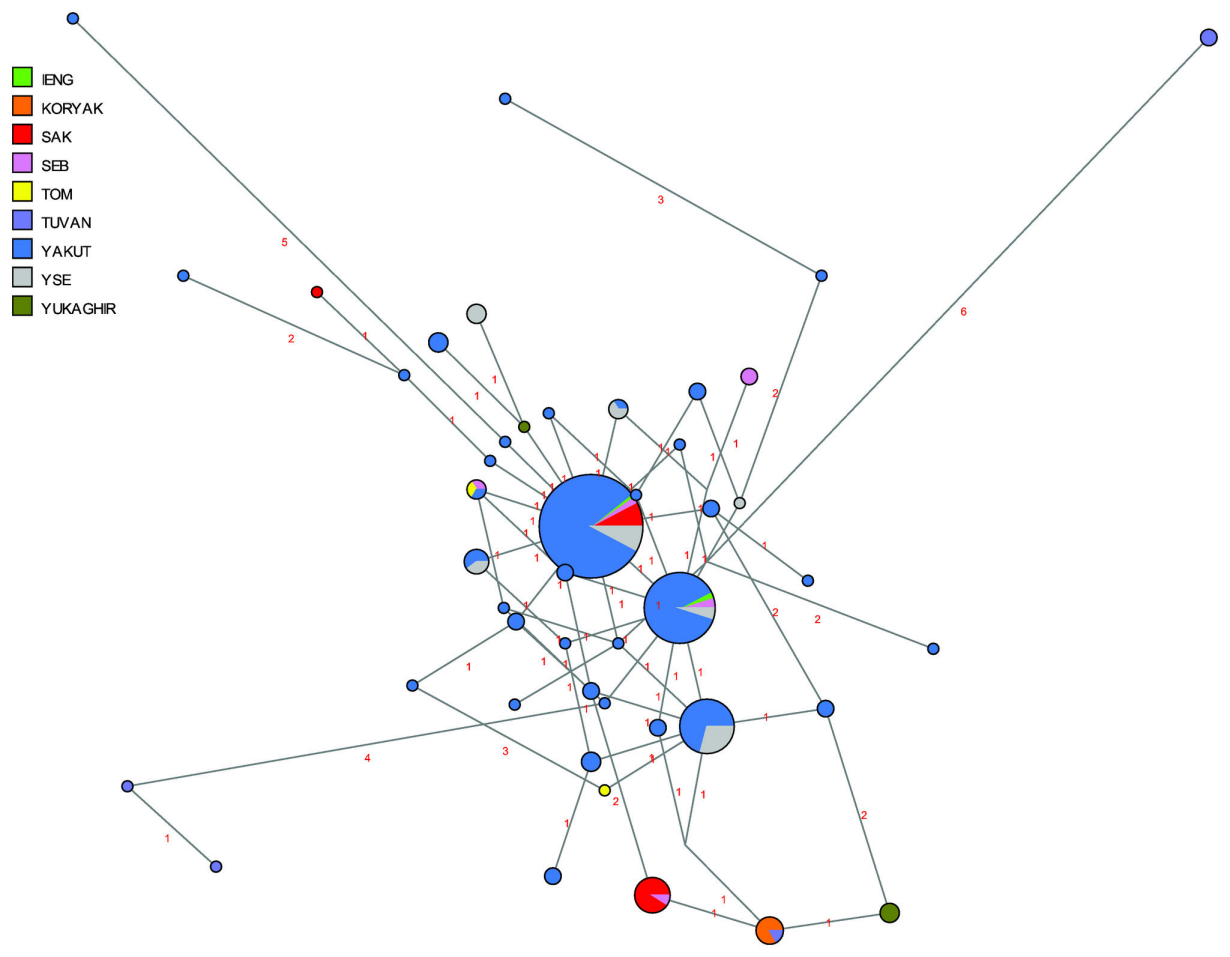
RM-MJ network of Y-chromosomal haplogroup N1c. Based on nine Y-STRs. The North Tungusic haplotypes are coloured by subgroup. The size of the nodes is proportional to the number of individuals carrying that node, and the number of mutations is indicated along the branches.

The network of STR haplotypes belonging to haplogroup N1c is highly complex with several multidimensional cycles (Figure 10). As seen previously [15], the network is dominated by haplotypes found in the Yakuts and the Yakut-speaking Evenks. The most common Yakut haplotype is shared by seven Sakkyryyr and two Sebjan Evens as well as one Iengra Evenk; the second most common Yakut haplotype is shared by two Sebjan Evens and one Iengra Evenk. A further haplotype is shared by one Yakut, one Tompo and one Sebjan Even. A small portion of the network completely lacks Yakut individuals; this is removed from the closest Yakut haplotypes by at least two mutational steps. This contains a haplotype found in one Sebjan and 10 Sakkyryyr Evens, a haplotype shared by Koryaks and Tuvans, and a Yukaghir haplotype. Thus, the network analysis of haplogroup N1c demonstrates that both the Iengra Evenk haplotypes are shared with Yakuts, as are one out of two Tompo Even, five out of eight Sebjan Even, and seven out of 18 Sakkyryyr Even haplotypes. Furthermore, one Tompo and one Sakkyryyr Even haplotype differ by only one mutational step from Yakut haplotypes. 

## Discussion

### Relationships of Tungusic populations

The major hypotheses about the origins of the North Tungusic Evenks and Evens postulate either a deep split from the Tungusic peoples of the Amur-Ussuri region and a fairly ancient divergence of Evenks and Evens [4] or a much shallower divergence of Evenks and Evens and a fairly recent split of the Northern Tungusic and Amur Tungusic populations [5,6]. The results of the current investigation show that while the Evens and Evenks indeed stem from a common ancestral population, genetic drift and differential admixture with neighbouring but unrelated populations have played a role in shaping their diversity, as was already found in a previous study [15]. 

The shared paternal ancestry is apparent in the high frequencies of Y-chromosomal haplogroup C3c1 which characterise these populations, cf. [15,17,22,23]. The shared maternal ancestry of Evenks and Evens is evident in the presence at relatively high frequency of mtDNA haplogroups C4a, C4b, and D4, which leads to a lack of differentiation of most subgroups in the CA plot, and especially in the non-significant and negative between-group variance in the AMOVA analysis comparing Evenks with Evens. The effect of drift can be seen in the low mtDNA diversity in these populations as well as by their scattered location in the MDS analysis. The effect of gene flow is evident in the affinities of Evenks with Yakuts, and Evens with Yukaghirs emerging in the networks of mtDNA haplogroups C4a and C4b and the haplotype sharing analysis, as well as in the sharing of haplotypes belonging to Y-chromosomal haplogroup N1c with Yakuts. 

These differential effects of drift and admixture have resulted in significant differences between individual Evenk and Even subgroups, as demonstrated by the AMOVA analysis (Table 2). Surprisingly, these differences are larger among the Evenk subgroups than among the Even subgroups, even though the latter are more widely dispersed geographically. Together with the significant correlation between geographic and genetic distances for four of the Even subgroups (Table 3), this might be an indication that the break-up of the ancestral Even population occurred at a later time than that of the Evenk ancestral population, possibly as a direct result of the Yakut expansion. As for the paternal lineages, although all North Tungusic subgroups have high frequencies of Y-chromosomal haplogroup C3c1, there is no sharing of C3c1 haplotypes between Evenks and Evens, indicating a fairly deep split between these populations and lack of subsequent admixture (Figure 8). Using the rho statistic [27] implemented in Network and a mutation rate of 0.0025 per locus per generation [28], we estimate the age of the STR diversity shared by Evenks and Evens to be 1916 ± 985 years. The age of the STR diversity specific to Evens is estimated to be 615 ± 418 years, that specific to Evenks 303 ± 177 years. The split of the Even and Evenk populations is thus estimated to have taken place between approximately 1900 BP and 500 BP – an estimate which brackets both the time of divergence proposed by Vasilevich [4] (~1500 BP) and that proposed by Tugolukov [5] and Janhunen [6] (sometime after 800 BP and possibly as recently as 400 BP). In a study of haplogroup C Y-chromosomes in northern Asia, Malyarchuk et al. [29] date the split between the Evenk and Even haplotypes to either 1400 (± 1060) or 390 (± 290) years ago, depending on whether the evolutionary or the pedigree-based mutation rate is used. The question of which mutation rate to use is still not settled, but it has been suggested for mtDNA data that it is preferable to use the pedigree rate in studies of relatively recent history, while the phylogenetic rate is preferable in studies of deep history [30,31]. Furthermore, a previous study of Yakut origins using the same set of Y-chromosomal STRs [14] found that the age resulting from the pedigree-based mutation rate was more compatible with linguistic, historical, and archaeological data for the origins of the Yakuts. Taking these circumstantial arguments into account lends greater weight to the younger date estimated by Malyarchuk et al. [29] and thus favours the late split between the Evenks and Evens suggested by Janhunen [6] and Tugolukov [5], and contradicts the early split proposed by Vasilevič [4]. 

As to the Tungusic populations of the Amur-Ussuri region, in the maternal line the Udegey appear quite distinct from their linguistic relatives the Evenks and Evens with respect to their haplogroup composition (Table S1). Nevertheless, although the second dimension of an MDS analysis separates the Udegey from the Siberian populations, in the first and third dimension they cluster with Yakuts and the Iengra Evenks (Figure S4). Similarly, in the AMOVA analysis, less than 6% of the variance is due to differences between the South Tungusic Udegey and the North Tungusic Evenks and Evens, in contrast to nearly 7% of the variance found among the different North Tungusic subgroups (Table 2), indicating that there are some affinities of the Udegey with their linguistic relatives. A direct link between the Udegey and the Evenks is evident in mtDNA haplogroup C4b: as shown in the network (Figure 3), the Udegey haplotypes are derived from a sequence type that is shared with all the Evenk subgroups. Links between the Udegey and southern Siberian populations are also apparent in the presence of haplogroup M7 and M9 (Table S1, Figure 4). These are found at low frequency in Mongolic-speaking populations, Tuvans from southern Siberia, and the Ulchi, a South Tungusic population of the Amur river; furthermore, M7 is found in Koreans with a frequency of nearly 10% [10,32]. The high frequency of haplogroup N9b and the presence of haplogroup Y1a, however, connects the Udegey with other populations of the Amur-Ussuri region rather than Siberia, as also shown in another study: in a PC analysis based on frequencies of mtDNA subhaplogroups the Udegey cluster with the Nivkh and the South Tungusic Ulchi of the Amur-Ussuri region, as well as, at a slight distance, with the North Tungusic Negidal and the Kamchatkan Koryaks and Itelmen [10]. This might indicate that the Udegey resulted from admixture between indigenous populations of the Amur-Ussuri region, such as those represented by the prehistoric “Okhotsk people” [33], and Tungusic-speaking immigrants from southern Siberia. This hypothesis of large-scale mixing in the prehistory of the Udegey is further confirmed by their combination of very low sequence diversity, which is as low as that found in the Nivkh, with intermediate levels of nucleotide diversity (Table 1). Alternatively, the shared haplotypes found in the Evenks and Udegey might be retentions from an earlier shared ancestral population; this would be in accordance with the proposed origin of the Tungusic languages and populations in the Amur region [6].

### Gene flow in the history of individual North Tungusic subgroups

With respect to the individual North Tungusic subgroups, the Taimyr Evenks stand out in showing connections in the maternal line with populations far to the east, notwithstanding their settlement in northwestern Siberia. Thus, two individuals carry a sequence type belonging to mtDNA haplogroup A2a, which is practically absent in western and central Siberia, but found in high frequency in the Siberian Eskimos and Chukchi and in low frequency in Koryaks and Chuvantsi, all populations settled far to the east of the Taimyr Peninsula [10,34]. Another haplogroup that links the Taimyr Evenks with more easterly populations is Y1a, present in 12.5% of the Taimyr Evenks, but in no other Evenk subgroup of our sample. This haplogroup is found in very high frequency in the Nivkh (65.8%); it is also found in the Udegey and in the eastern Even subgroups Kamchatka and Berezovka (Table S1). It is furthermore present in high frequency in the Ulchi and Negidal, Tungusic populations of the Amur-Ussuri region and in 9%-10% in eastern Evenks as well as Koryaks from Kamchatka [10,20,32], as well as in low frequency in Central and Vilyuy Yakuts [17] (Table S1). How the presence of these haplogroups of far eastern provenance can be explained among the Taimyr Evenks is not clear; they are not found in their neighbours on the Taimyr Peninsula, the Turkic-speaking Dolgans or the Samoyedic Nganasan [10,17]. In their paternal lineages, the Taimyr Evenks do not differ from the Stony Tunguska Evenks, who are settled to their south: they have high frequencies of haplogroups C3c and N1b, and share three out of five C3c-STR haplotypes with the Stony Tunguska and Iengra Evenks and one of their two N1b-STR haplotypes. In addition, they show evidence of European admixture in the paternal line: three individuals carry haplogroup R1a (Table 4).

The Iengra Evenks are settled in the south of Yakutia in a region where intense contact and intermarriage between Yakuts and Evenks has been recorded [35], and they show clear evidence of Yakut admixture in both the paternal and the maternal line, sharing two Y-chromosomal N1c-STR haplotypes and two out of three mtDNA haplogroup D5a2a2 sequences with Yakuts. Although they are separated in the second dimension of the MDS analysis, probably in result of genetic drift [15], their close maternal affinities with their linguistic and geographic neighbours, the Nyukzha Evenks, are evident in the four haplotypes (31% of the Iengra mtDNA haplotypes) shared exclusively with the latter (Table S2).

The Sakkyryyr Evens also show evidence of Yakut admixture in both the maternal and the paternal lines. Thus, rather than clustering with their closest geographic and linguistic neighbours, the Sebjan Evens, they are pulled towards the Yakuts in the MDS and CA plots, and they share nine out of their 19 mtDNA haplotypes (47.4%) with the Yakuts, four of these uniquely (Table S2). Two of these belong to haplogroup F1b and one to haplogroup M7, which are characteristic of populations of southwestern and southern Siberia rather than those of northeastern and central Siberia [10,18,32,36–38]. Furthermore, one shared haplotype belongs to haplogroup D5a2a2, which is characteristic of the Yakuts. This indicates that the direction of gene flow in these cases is likely to have been from Yakuts – who retain a signature of their southern origins [14,17] – into the Sakkyryyr Evens. This Yakut maternal admixture might explain the high sequence and nucleotide diversity values found in the Sakkyryyr Evens in comparison to other North Tungusic subgroups (Table 1). Likewise, the network analysis of Y-chromosomal haplogroup N1c (Figure 10) provides a clear indication of gene flow from Yakuts into the Sakkyryyr Evens, in that seven STR haplotypes are shared with Yakuts, with an additional haplotype separated by only one mutational step. Furthermore, a haplotype that was not included in the analysis because of a duplication in DYS393 is also shared with Yakuts (with the exception of the duplication [15]). Thus, 32% - 36% of the Sakkyryyr Even Y-chromosomes are most likely to be of Yakut origin. This indicates that the Sakkyryyr Even population incorporated entire Yakut communities, both men and women, during their history, and this might partly explain why the Sakkyryyr Evens have given up their North Tungusic language in favour of Yakut.

In contrast, the neighbouring Sebjan Evens do not show evidence of substantial amounts of Yakut gene flow in the maternal line: they are located at a distance from the Yakuts in the MDS plot and cluster with the Taimyr Evenks and the Tompo Evens in the CA analysis. Furthermore, they share only three of their eight haplotypes (37.5%) with Yakuts, none uniquely, and two of these shared haplotypes belong to haplogroups C4a1c and D4l2 characteristic of North Tungusic populations (Table S2). These shared haplotypes found in the Sebjan Evens might therefore be due to maternal Tungusic admixture into Yakuts during the expansion of the latter [14]. However, the proportion of Yakut male admixture in the Sebjan Evens is higher than that detected in the Sakkyryyr Evens: five Y-STR haplotypes belonging to haplogroup N1c are directly shared with Yakuts, and two more are separated from a common Yakut haplotype by two mutational steps. Furthermore, a haplotype not included in the network analysis because it contained a duplication at DYS393 is also shared with Yakuts (again excluding the duplication [15]). This indicates that approximately half (43% - 57%) of the Sebjan Even paternal genepool is of Yakut origins and indicates male-biased admixture from Yakuts to Evens rather than the incorporation of both maternal and paternal lineages detected in the Sakkyryyr Evens. Interestingly, although the Sebjan Evens have retained their Even language, this has undergone striking changes under the influence of Yakut [39].

The Tompo Evens are close to Yukaghirs in the MDS analysis based on mtDNA sequences, as was seen previously [15] (where this subgroup was called Central Evens). However, only two haplotypes are shared between Tompo Evens and Yukaghirs, and these are also shared with Stony Tunguska, Nyukzha, and Iengra Evenks and Kamchatkan Evens (Table S2). In the paternal line, the Tompo Evens are characterized by high frequencies of Y-chromosomal haplogroup N1b (Table 4), with all the individuals belonging to this haplogroup carrying a STR haplotype that is shared with Evenks. This haplogroup is found at highest frequency in the Samoyedic-speaking populations of northwestern Siberia, the Nganasan, Enets, and Nenets [22,24], and in high frequency in some Turkic-speaking populations of southern Siberia [26]. It is furthermore characteristic of North Tungusic populations of western Siberia, but is absent in those of the east: it is found in 39% in the Taimyr Evenks and 27.5% in the Stony Tunguska Evenks (Table 4), in 16.7% in a sample of ‘Western Evenks’ and in 24.4% in a geographically undefined sample of ‘Evenks’, but in only 2.5% in Chinese Evenks and is absent in Eastern Evenks, Evens, and Oroqen, who are closely related culturally and linguistically to Evenks [22,26]. The presence of this haplogroup in the Tompo Evens therefore appears indicative of paternal gene flow from Evenks. However, it is intriguing that this Evenk admixture led only to an incorporation of Y-chromosomal haplogroup N1b in the Tompo Evens, without any C3c1 Y-chromosomes being transferred. Possibly this presumed admixture event was restricted to one individual, with subsequent expansion of his Y-chromosomal lineage, as indicated by the fact that all the Tompo Evens carrying haplogroup N1b share the same STR haplotype.

The mtDNA haplogroup composition of the eastern Even subgroups, Berezovka and Kamchatka, with substantial frequencies of the characteristic eastern Siberian and Kamchatkan mtDNA haplogroups G1, Y1a and Z [10,20], indicates a certain amount of admixture with local populations during their eastward expansion. In contrast, their Y-chromosomal genepool consists solely of haplogroup C3c1, which is typical of North Tungusic populations, indicating that these interactions with local groups were biased towards the female line [15]. On Kamchatka, although the Evens have 15.4% of mtDNA haplogroup G1, which is found in very high frequencies in Kamchatkan Koryaks and Itelmen [20], there is no direct evidence for recent gene flow from Koryaks into the Even population. In contrast, gene flow from Evens into Koryaks can be shown to have taken place, both in the presence of a shared sequence type belonging to mtDNA haplogroup D4 and in the sharing of a Y-chromosomal haplotype belonging to haplogroup C3c1. 

### Demographic history of the populations

The mtDNA diversity values and Bayesian Skyline analyses (Table 1, Figure 7, Figure S6) show substantial differences in demographic history between the populations included in this study. The Yakuts stand out as having higher diversity values than most of the other populations; this is in good accordance with their much larger census population size in comparison to that of the other populations (478,000 Yakuts vs 38,000 Evenks, 21,000 Evens, 1500 Udegey, 1600 Yukaghir, 4600 Nivkh, and 7900 Koryaks in 2010 [40]). They furthermore show an increase in population size approximately 1000 years BP (Figure 7A); this fits with previous estimates based on the Y-chromosome and mtDNA [14,41] and is also in relatively good accordance with archaeological data showing that the ancestors of the Yakuts migrated to the north in the 13^th^ or 14^th^ centuries CE [42,43]. The Evenk and Even subgroups generally have lower diversity values than the Yakuts, with the exception of the Sakkyryyr Evens. The Udegey, Yukaghirs, Nivkh, and Koryaks, who are traditionally predominantly sedentary hunter-gatherers and especially fishermen, all have relatively low mtDNA diversity values and basically flat BSP curves with a lower effective population size than that found in the Evenks and Evens and especially Yakuts. This pattern, which indicates that they have had a constantly low effective population size, differs from the pattern found in the North Tungusic populations, who not only have a higher effective population size, but where a slight increase is apparent before the recent decline. This might reflect the differences in life-style between the sedentary hunter-gatherers and the nomadic Evenks and Evens, whose exceptional mobility would have increased their pool of potential spouses. The decline in population size evident in the BSPs for the Evens and Evenks overlaps the increase appearing in the Yakuts; it is therefore possible that the northward expansion of the Yakuts resulted in a decrease in population size of the North Tungusic populations. 

## Conclusions

Our analysis of complete mtDNA genome sequences and Y-chromosomal SNP and STR variation has revealed a differentiated picture of the relationships among Tungusic populations. Although the Amur Tungusic Udegey are linguistic relatives of the North Tungusic Evenks and Evens, in the maternal line most traces of their possible genetic relationship have been erased by the effects of drift and admixture both in the Siberian populations as well as in the Udegey. As to the Evenks and Evens, while they retain evidence of their shared ancestry, admixture with neighbouring populations, especially in the maternal line, has led to their genetic differentiation. In-depth investigations of the autosomal variation in these populations will enable deeper insights into their population history and relationships among each other and with other populations of Siberia.

## Materials & Methods

### Ethics statement

The collection of the samples was approved by the Ethics Committee of the University of Leipzig, the Ethics Committee of the Research Centre for Medical Genetics, Russian Academy of Medical Sciences, Moscow, and the Institutional Review Board of the University of Kansas. Written and/or oral consent to use their samples was obtained from all donors after explanation of the aims of the study. The Institutional Review Board of the University of Kansas approved the substitution of oral informed consent in lieu of written consent in the case of the Udegey, Koryaks, and Kamchatkan Evens, since donors were suspicious of signing written statements. The oral consent was witnessed by the Russian team members of the sampling expedition.

### mtDNA analyses

We generated a total of 525 full genome mtDNA sequences from 130 Evenks belonging to four subgroups (from northwest to southeast: Taimyr, Stony Tunguska, Nyukzha, and Iengra), 122 Evens belonging to five subgroups (from west to east: Sakkyryyr, Sebjan, Tompo, Berezovka, and Kamchatka), 31 Udegey, 169 Yakuts belonging to three subgroups (Vilyuy, Central, and Northeast), 20 Yukaghirs, 15 Koryaks, and 38 Nivkh (Figure 1) using the method described in the Supplementary Materials of Barbieri et al. [44]. The Taimyr Evenk samples were collected in the village of Khantayskoe Ozero, the Sebjan and Berezovka samples in the villages of Sebjan-Küöl and Berezovka (Yakutia), respectively, the Kamchatkan Evens and Koryaks in the villages of Esso and Anavgai, the Nivkh were collected in northern Sakhalin, and the Udegey were collected in the village of Gavsjugi (Khabarovsk Region). For details on the sample locations for the other (sub)populations see 14,15. 

The sequences were generated with an Illumina Genome Analyzer IIx sequencer to an average coverage of 274x and full sequences were deposited in GenBank under accession numbers KF148067-KF148359 and KF148361-KF148592. Three different alignments were generated for analysis. For calculation of pairwise Φ_ST_, standard mtDNA diversity indices, and Analysis of Molecular Variance (AMOVA) in Arlequin v3.5 [45] all indels, positions with unclear and missing data (coded as N’s) and the poly-C region (16183 - 16194) were removed, leaving an alignment of length 16,507 bp. For the analysis of haplotype sharing using an in-house Python script as well as Bayesian Skyline Plots (BSP) produced with BEAST, a second alignment was generated which omitted only the poly-C region and positions containing Ns, but included all indels, resulting in an alignment length of 16,521 bp. The third alignment was used to construct Median-Joining networks; it was 16,478 bp in length to accommodate published data downloaded from GenBank. Haplogroups were assigned using the online tool Haplogrep [46] in reference to PhyloTree Build 15 [47], using rCRS [48] as a reference. 

For each population and subgroup the ideal substitution model was calculated using jModelTest v 2.1 [49]. With the information as to the appropriate substitution model, each (sub)population was then tested for adherence to a molecular clock in MEGA v5 [50]; for none of the (sub)populations was the null hypothesis of a molecular clock rejected. Bayesian Skyline plots (BSP) were then generated using the BEAST package v1.6 [51,52], partitioning the data between the coding (577 - 16,023) and non-coding (16,024 – 576) regions and applying the mutation rates from Soares et al. [53] (1.708E^-8^ and 9.883E^-8^, respectively), using a strict clock model.

Median Joining (MJ) networks [54] were generated using the Network 4.6 and Network Publisher v1.3 programmes (www.fluxus-engineering.com), with transversions given a threefold higher weight than transitions. Correspondence analysis (CA) and multi-dimensional scaling (MDS) plots were generated with STATISTICA v10 [55]. Mantel tests of the correlation between geographic great-circle distances – calculated with the R package “geosphere” [56] – and Φ_ST_ genetic distances between (sub)populations were performed in R using the “ade4” package [57].

Published mtDNA genome sequences were downloaded from GenBank for inclusion in the network analyses; these included additional Yukaghir, Evenk, Even, Udegey, Koryak, and Yakut sequences as well as Mongolic-speaking Buryats and Khamnigans, Turkic-speaking Tofalar, Tubalar, Altai-Kizhi, Shor, Teleut, Tuvan, and Kazakh from South Siberia, Kets, who speak an isolate language, and Finno-Ugric-speaking Mansi from western Siberia, Samoyedic-speaking Nganasan from the Taimyr Peninsula, Tungusic-speaking Negidal and Ulchi from the Amur-Ussuri region, Chukchi from Chukotka who speak a Chukotko-Kamchatkan language, and Eskimos [10,16,18,32,34]. 

### Y-chromosomal analyses

To investigate the paternal relationships of the Evenks and Evens, Y-chromosomal SNPs were genotyped in the Sebjan, Berezovka, and Kamchatkan Evens as well as in the Taimyr Evenks following the protocol of de Filippo et al. [58]; subhaplogroups of C and N were defined by genotyping the SNPs M48, M86, and P43 following the method of Nasidze et al. [59] and Pakendorf et al. [14], and were named according to the nomenclature of Karafet et al. [60]. In addition, 12 short tandem repeat (STR) loci were genotyped using the Promega Y-Powerplex kit (http://www.promega.com). The newly generated Y-chromosomal genotypes were compared to previously published Y-chromosomal data [14,15,61]; for this purpose, the Even individuals were reassigned from the previous subdivision of Western and Central Evens [15] into the subdivision followed here, namely Sakkyryyr, Sebjan, and Tompo. While the Y-STR data published by Rubicz et al. [61] were not associated with haplogroup information, this was obtained from Rubicz [62] for the Koryaks and Evens. For network analyses of STR haplotypes belonging to haplogroups C3c, N1b, and N1c only nine STRs overlapping between the datasets were used, and individuals with missing data or duplications at DYS393 were excluded. This concerned one Berezovka Even belonging to haplogroup C3c1 with missing data, one Sakkyryyr and one Sebjan Even belonging to haplogroup N1c with a duplication at DYS393, and two Tompo Evens belonging to haplogroup C3c1 with a duplication at DYS393. For the network analyses, the STRs were assigned weights inversely proportional to their variance within each haplogroup [63]. Since the network of haplogroup N1c was very complex with a large number of multidimensional reticulations, a joined Reduced Median and Median Joining analysis was performed. The newly generated Y-chromosome genotypes are available in Table S3.

The age of the Y-STR diversity accumulated in Evenks and Evens belonging to haplogroup C3c1 was estimated with the rho statistic [27] implemented in Network, using a mutation rate of 0.0025 per locus per generation [28]. Assuming a male generation interval of 30 years [64] and including nine loci, this amounts to one mutation every 1333 years.

## Supporting Information

Figure S1 **MJ-network of mtDNA haplogroup D4.** The North Tungusic haplotypes are coloured by population (Evenks and Evens) rather than subgroup. The size of the nodes is proportional to the number of individuals carrying that node, and the number of mutations is indicated along the branches. The haplotypes marked by arrows are discussed in the text.
(EPS)

Figure S2 **MJ-network of mtDNA haplogroup D5a2.** The North Tungusic haplotypes are coloured by subgroup. The size of the nodes is proportional to the number of individuals carrying that node, and the number of mutations is indicated along the branches.
(EPS)

Figure S3 **MJ-network of mtDNA haplogroup A.** The North Tungusic haplotypes are coloured by population (Evenks) rather than subgroup. The size of the nodes is proportional to the number of individuals carrying that node, and the number of mutations is indicated along the branches. Subhaplogroups discussed in the text are labelled.
(EPS)

Figure S4 **Dimensions 1 vs. 3 of a three-dimensional MDS analysis.** Based on pairwise Φ_ST_ values between populations; stress = 0.06. (Sub)population abbreviations as in Table 1, with colours distinguishing the different populations: green = Evenks, red = Evens, pink = Udegey, blue = Yakuts, olive = Yukaghirs, orange = Koryaks, black = Nivkh.
(EPS)

Figure S5 **Dimensions 2 vs. 3 of a three-dimensional MDS analysis.** Based on pairwise Φ_ST_ values between populations; stress = 0.06. (Sub)population abbreviations as in Table 1, with colours distinguishing the different populations: green = Evenks, red = Evens, pink = Udegey, blue = Yakuts, olive = Yukaghirs, orange = Koryaks, black = Nivkh.
(EPS)

Figure S6 **Bayesian Skyline Analysis plots.** Based on complete mtDNA genome sequences and a strict clock model. A: All Evenks, B: Nivkh, C: Koryaks, D: Yukaghir.
(EPS)

Table S1 **List of mtDNA (sub)haplogroups found in the dataset and their frequencies in individual (sub)populations.**
(XLS)

Table S2 **Analysis of shared haplotypes.**
(XLS)

Table S3 **Y-chromosomal genotypes generated for this study.**
(XLS)
